# Challenges and Uncertainties in the Diagnosis of Cardiac Amyloidosis: A Case Report

**DOI:** 10.7759/cureus.60954

**Published:** 2024-05-23

**Authors:** Alia A Ibrahim, Mohammed Gaffar Mohammed, Haitham B Elmasharaf, Ibrahim Y Osman, Nagoud M Ali

**Affiliations:** 1 Internal Medicine, Dr. Sulaiman Al-Habib Hospital - Al Sweidi Branch, Riyadh, SAU; 2 Rheumatology, Prince Sultan Military Medical City, Riyadh, SAU; 3 Cardiology, Prince Sultan Military Medical City, Riyadh, SAU; 4 Pathology, Prince Sultan Military Medical City, Riyadh, SAU

**Keywords:** chemotherapy cardiac amyloidosis, heart transplant cardiac amyloidosis, echocardiography findings cardiac amyloidosis, heart failure cardiac amyloidosis, atrial fibrillation cardiac amyloidosis, delayed diagnosis cardiac amyloidosis, imaging in cardiac amyloidosis, speckled tracking echocardiography, diagnostic criteria of cardiac amyloidosis, al cardiac amyloidosis

## Abstract

Amyloidosis is the condition when starch-like misfolded proteins form insoluble fibrils that deposit in tissues and cause dysfunction. Cardiac amyloidosis occurs due to the deposition of amyloid fibrils at the cardiac level and is an important cause of heart failure. This case reveals a patient with significant heart failure and arrhythmia, which later on turned out to be caused by cardiac amyloidosis. While regarded as a rare disease in practice, in retrospect, there are a lot of signs and imaging indicators, particularly in echocardiography that warrant an investigation of cardiac amyloidosis. In this case review, red flags in echocardiography that should endorse further testing for underlying cardiac amyloidosis are highlighted.

## Introduction

The word amyloid is of Greek and Latin origins (derived from *amylon* and *amylum*, respectively) and means starch-like. This term was co-opted from botany by Rudolf Virchow in 1854 to be used as a medical term known today as amyloidosis [1]. In amyloidosis, starch-like precursor proteins form insoluble fibrils that get deposited in tissues and cause dysfunction [2]. The International Society of Amyloidosis currently recognizes 36 types of amyloidosis, all of which are attributed to their corresponding underlying amyloidogenic precursor [3].

Cardiac amyloidosis (CA) is a progressive infiltrative disease that occurs due to the deposition of amyloid fibrils at the cardiac level [4]. Two forms of CA most commonly affect the heart: light-chain cardiac amyloidosis (AL-CA) and transthyretin cardiac amyloidosis (ATTR-CA) [5]. Distinguishing these two types of CA is pivotal, as their clinical course and management are vastly different [6]. Currently, the prevalence of CA in the Middle East is noticeably underrepresented in literature and the condition remains underdiagnosed, with the vast majority of patients going undiagnosed [7]. 

The cardinal symptoms of CA include fatigue, shortness of breath, orthopnea, exercise intolerance, and peripheral edema; rarer symptoms include syncope, arrhythmia, and sudden cardiac death [8]. CA classically presents as heart failure with preserved ejection fraction (HFpEF) [9]. When assessing CA, multiple extra-cardiac symptoms and signs must also be considered [10]. If untreated, ATTR-CA carries a mortality risk in two to six years post-diagnosis, whereas AL-CA carries a worse prognosis, with a mortality risk in less than six months [10,11].

The position paper of the European Society of Cardiology Working Group on Myocardial and Pericardial Diseases contends that both invasive and noninvasive investigations can be implemented depending on the underlying type of CA. The position paper focuses on two distinct management areas: disease-modifying management, which focuses on suitable chemotherapy regimen algorithms, and treatment of complications and comorbidities [4].

The success of orthotopic heart transplantation (OHT) and sequential autologous stem cell transplantation (ASCT) has revived enthusiasm for heart transplantation for patients with end-stage CA. OHT outcomes depend on many factors, including careful patient selection, effective plasma cell-directed therapies before and after transplantation, and improvement in pharmacologic therapy options. The extremely high mortality rate observed in those on the heart transplant waiting list has led to changes to the heart allocation system, granting CA patients a higher status (i.e., status 4) [12]. Pertaining to the timing of ASCT post-OHT, various works support undergoing ASCT six months post-OHT, but some argue waiting for one year post-OHT to perform ASCT leads to improved survival rates [13].

## Case presentation

**Figure 1:**
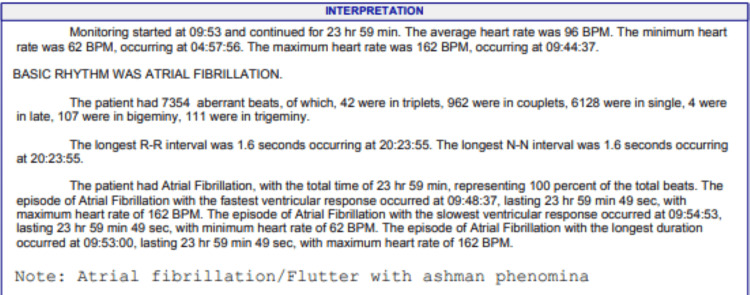
24-hour Holter interpretation showing atrial fibrillation with rapid ventricular response as well as Ashman phenomenon.

**Figure 2:**
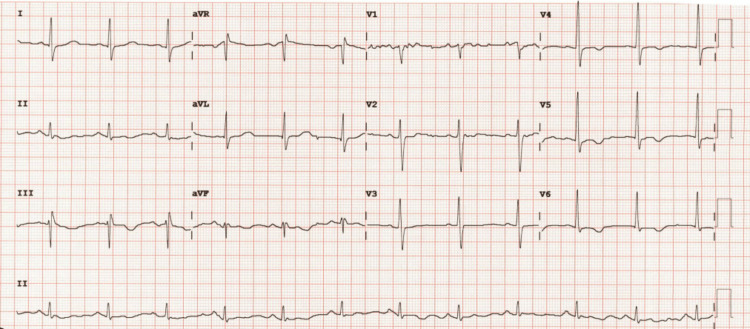
Electrocardiogram showing T wave changes in inferior, lateral, and anterior leads.

**Figure 3:**
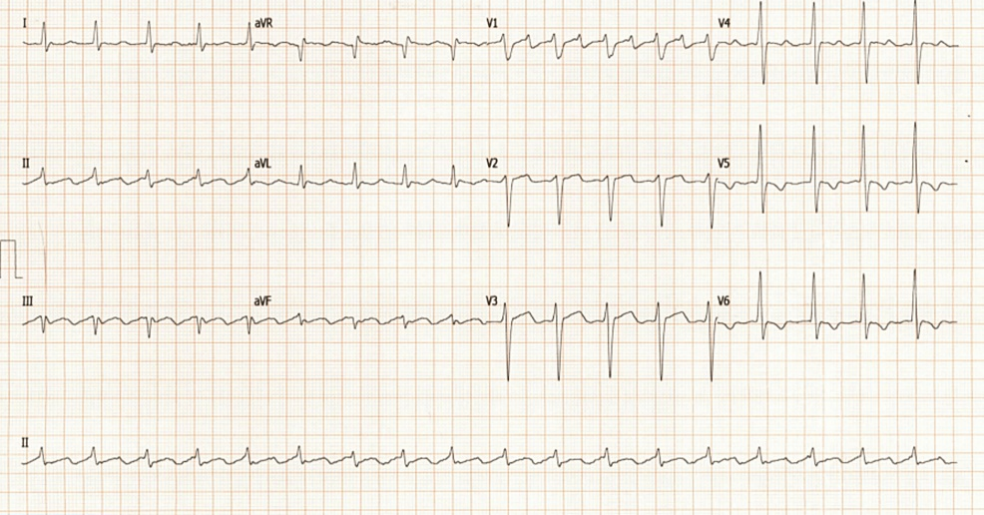
Electrocardiogram showing focal atrial tachycardia.

**Figure 4:**
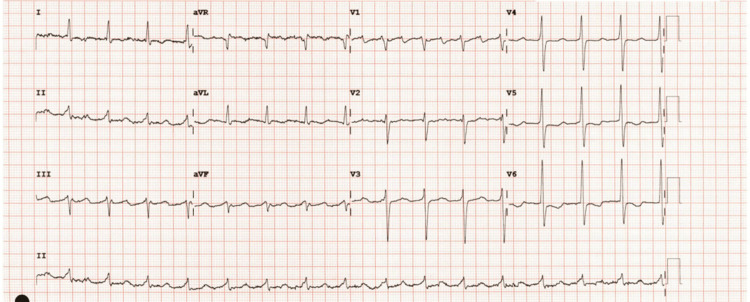
Electrocardiogram showing multiple premature atrial complexes and left ventricular strain pattern (V5 and V6).

A 69-year-old male known to have hypertension, type 2 diabetes, and mild dyslipidemia developed new-onset palpitations and dyspnea on exertion. He subsequently developed orthopnea, followed by dyspnea at rest, which would be exacerbated by acute attacks of severe dyspnea. These attacks of acute dyspnea became more frequent, occurring up to two times per week, resulting in urgent visits to the emergency room. Chest X-rays usually showed pulmonary edema. Around this time, he was also noted to have developed bilateral large leg swellings and began to have palpitations. Interpretation of a 24-hour Holter monitoring revealed atrial fibrillation, which can be seen in Figure 1. Other electrocardiography (ECG) findings include T wave changes, focal atrial tachycardia, and premature atrial contractions, as seen in Figures 2-4. He was subsequently started on Guideline Directed Medical Therapy for Heart Failure, as well as rate control and anticoagulation medications. These were furosemide 80 mg per oral (PO) divided with instructions according to symptoms, metoprolol succinate 50 mg PO once daily (OD), spironolactone 12.5 mg PO OD, valsartan 80 mg PO OD, empagliflozin 10 mg PO OD, and rivaroxaban 20 mg PO OD.

**Figure 5:**
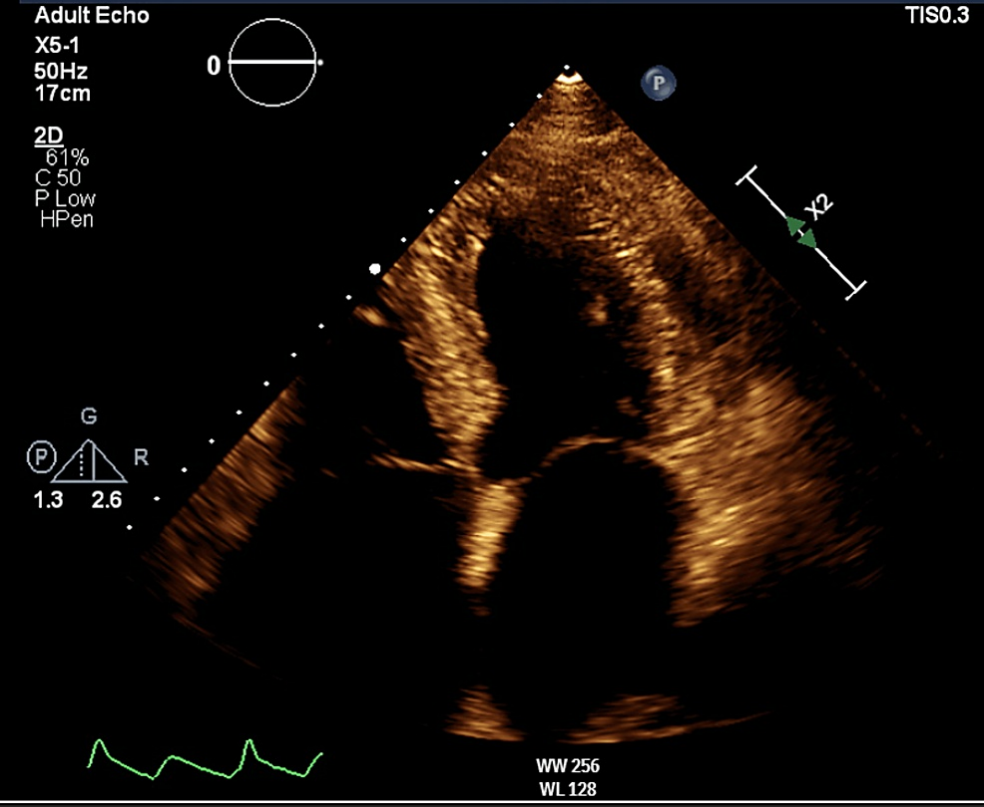
Two-dimensional four-chamber transthoracic echocardiogram (TTE) showing granular speckling, left ventricular hypertrophy, and severe biatrial dilatation.

**Figure 6:**
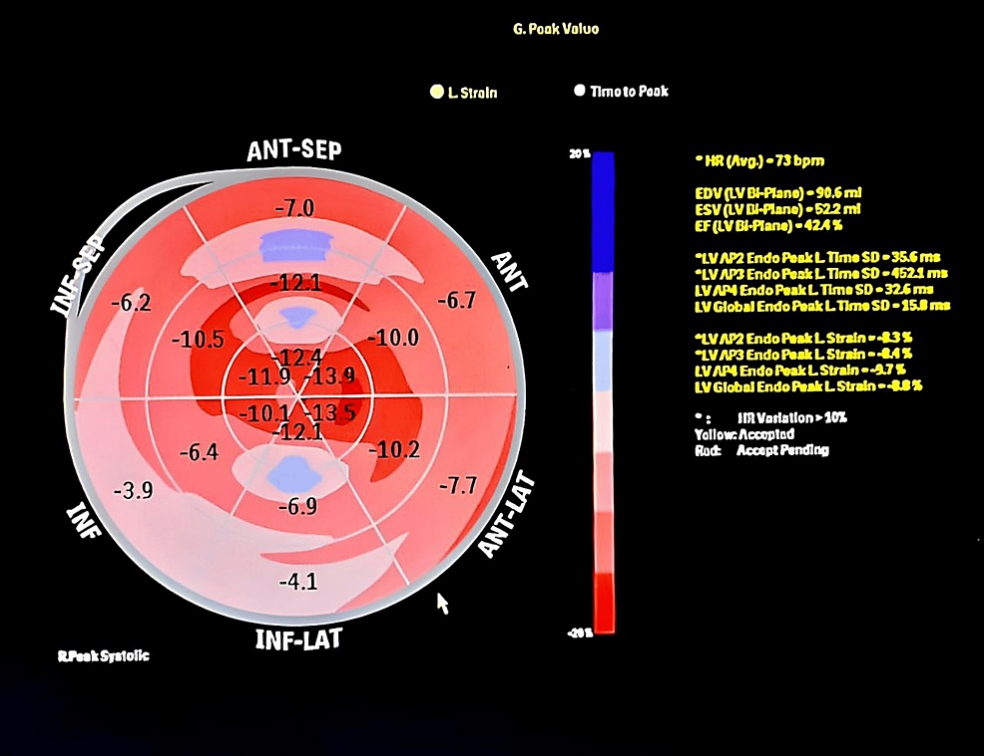
Two-dimensional speckle tracking echocardiography bull’s eye plot showing left ventricular strain and apical sparing.

**Figure 7:**
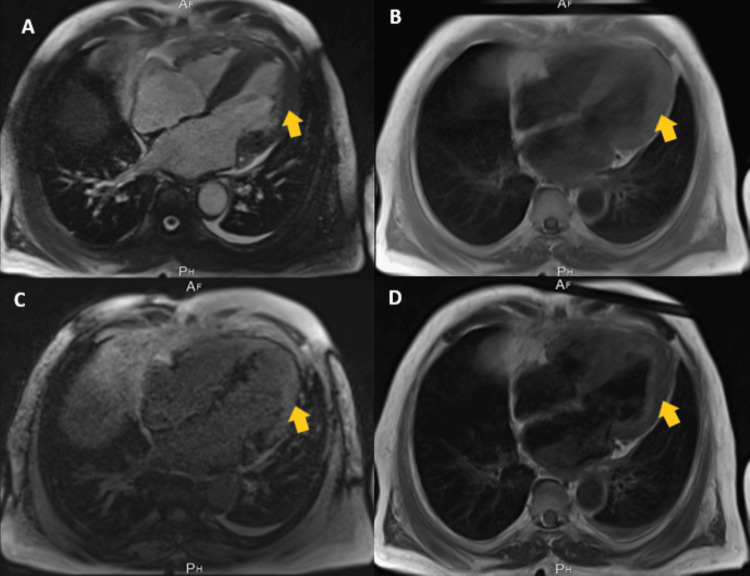
Four-chamber cardiac magnetic resonance (CMR) imaging: (a) Cine, (B) T1, (c) magnitude-corrected T1, and (D) T2 showing concentric left ventricular hypertrophy.

**Figure 8:**
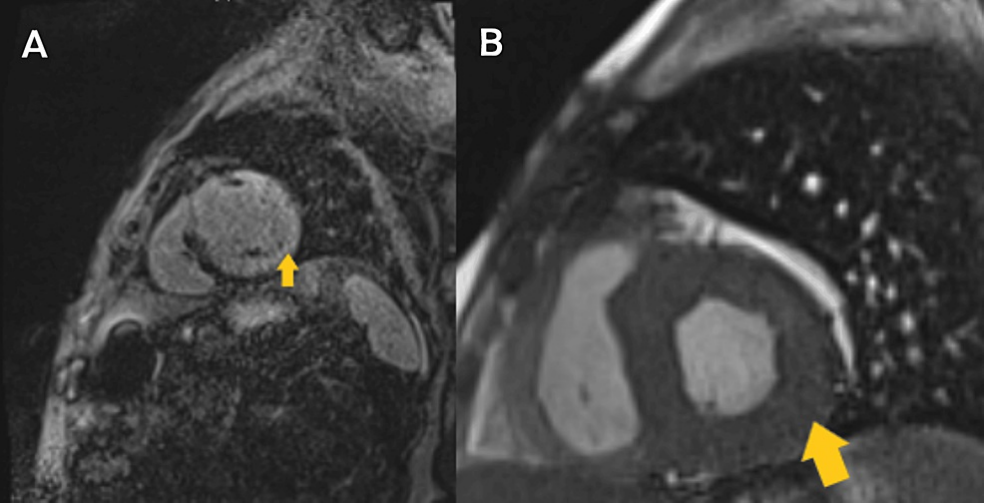
Cardiac magnetic resonance imaging - sagittal view (a) diffuse subendocardial late gadolinium enhancement and (b) basal short-axis steady-state free precession (SSFP) concentric left ventricular hypertrophy.

**Figure 9:**
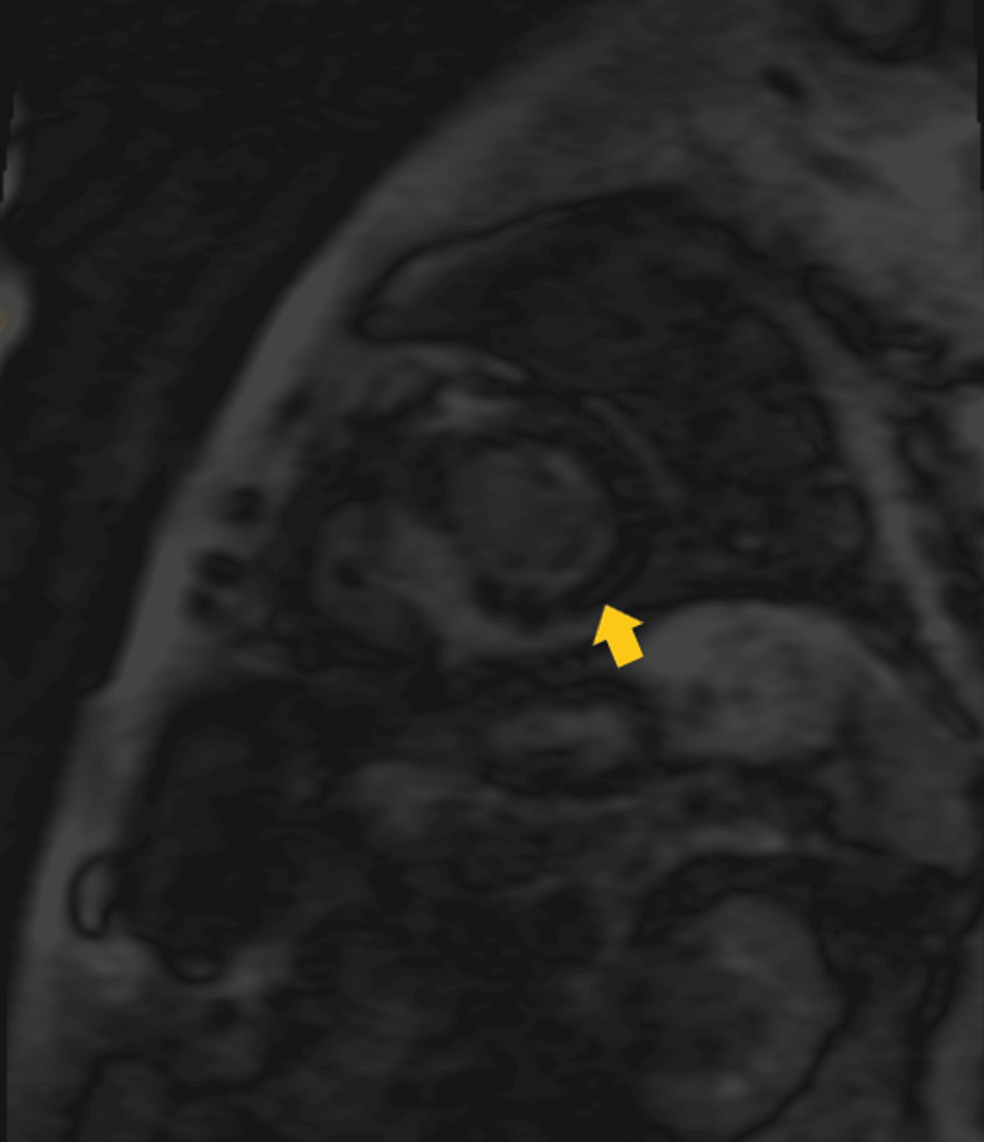
Cardiac magnetic resonance imaging (T1 Scout) with the yellow arrow showing early nulling of the myocardium and a lack of homogenous nulling.

**Figure 10:**
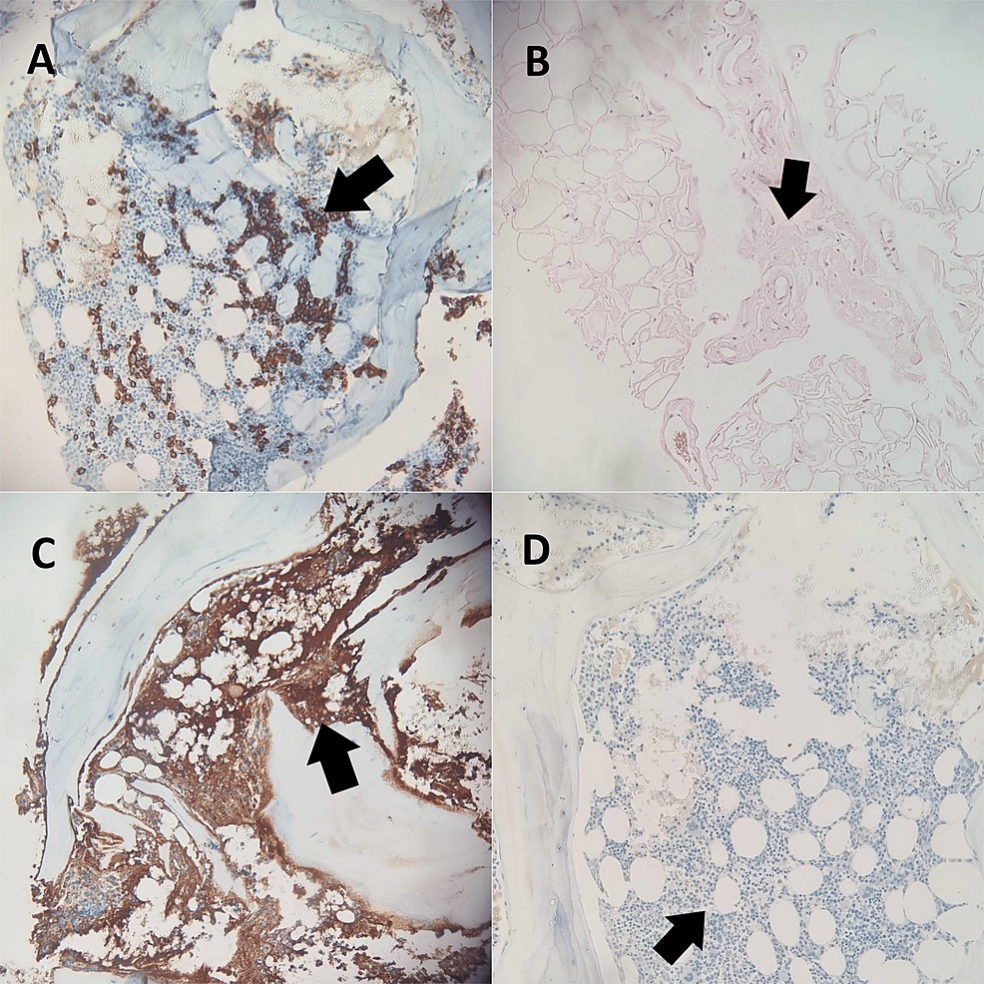
Microscopy studies: (a) CD 138 plasma cells, (b) polarized hematoxylin and eosin stain with black arrows indicating amyloid, (c) positive lambda light chains, and (d) negative kappa light chains.

**Figure 11:**
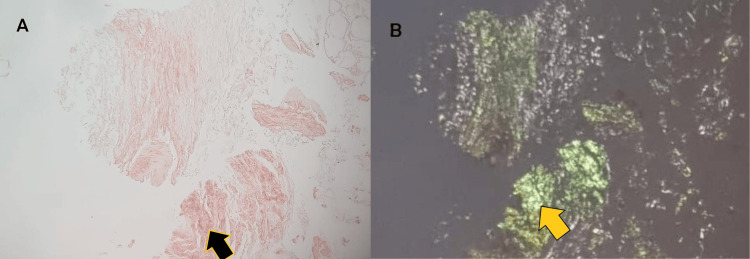
Microscopy studies: (a) fat Congo red special stain (black arrow) at high power (amorphous eosinophilic extracellular material); (b) same slide as (a) but with positive apple green birefringence (yellow arrow) under polarized light microscope..

Within more than two years from the onset of symptoms, restrictive pathology was suggested after reviewing various two-dimensional echocardiography tests, which demonstrated granular speckling, left ventricular hypertrophy, and severe bi-atrial dilatation, as seen in Figure 5. Video 1 shows two-dimensional echocardiography exhibiting left-sided hypokinesis as the disease progressed. These results prompted further specific imaging, including the two-dimensional speckled tracking echocardiogram (STE) seen in Figure 6, which showed typical left ventricular strain and apical sparing. A cardiac magnetic resonance (CMR) showed left ventricular hypertrophy of >15 mm and further confirmed a restrictive cardiomyopathy (RCM) with an infiltrative pattern of filling defects on gadolinium enhancement among other findings all of which are shown in Figures 7-9. A cardiac Tc99m pyrophosphate single-photon emission computed tomography (cardiac PYP SPECT) suggested AL-CA as opposed to early ATTR-CA, after which comprehensive hemato-oncological testing was done, the results of which are given in Table 1. Microscopy findings are presented in Figures 10, 11.

**Video 1:** Four-chamber transthoracic echocardiogram (TTE) showing hypokinesis of the left ventricle and ventricular septum.

**Table 1:** Results from diagnostic tests performed in October 2019.

Test	Findings
Bone marrow aspirate (iliac)	Active trilineage hematopoiesis with some dysplastic changes and plasma cell infiltration
Bone marrow biopsy (iliac)	Clonal plasma infiltrate estimated at 40%–60% expressing CD138, and lambda light chain reticulin stain showing (I-II) fibrosis
Intra-abdominal fat biopsy	Extensive amyloid deposit and positive Congo red stain
Nuclear medicine bone scan	Suggestive of either AL-CA or early ATTR-CA
Whole-body positron emission tomography scan	No bone metabolic activity lesions
Hematological tests	Monoclonal lambda light chains = 147 mg/L (5.7–26.3 mg/mL); lambda/kappa light chain ratio 0.05% (0.26%–1.65%)
Urine test for Bence–Jones proteins	Detected
Immunoglobulin G	32.2 g/L (6–16 g/L), others were low

 

Based on the above-mentioned results, in addition to serum protein electrophoresis with immunofixation, urine protein electrophoresis with immunofixation, and distinctly higher lambda light chains, the patient was diagnosed with AL-CA. For cardiac markers, N-terminal prohormone of brain natriuretic peptide and troponin-T were regularly followed during the course of his disease, and the patient remained negative for troponin-T throughout. Coronary angiography was performed but did not reveal anything of significance.

The patient was started on cyclophosphamide, bortezomib, dexamethasone, and prolia, and planned for a total of 16 cycles. After six months, daratumumab was added. The response was adequate at first but slowed down as the disease progressed. The patient also suffered various side effects including two osteoporotic fractures. In 2022, it was decided that the patient should undergo OHT as soon as feasible due to disease burden and the age limit precipice for transplant surgery. 

## Discussion

This case highlights diagnostic obstacles, as well as certain red flags for CA in echocardiography, and its limitations. In this case, CMR imaging and cardiac PYP SPECT played significant roles in diagnosing CA.

Diagnostic setbacks

The patient, who was a 69-year-old male known to have hypertension, type 2 diabetes, and mild dyslipidemia, had a myriad of manifestations starting from late 2017 well into late 2019. In late 2019, after a cumulative revision of the patient’s symptoms and signs, the decision was made to explore infiltrative causes of his HF, with the most common infiltrative heart disease outside of sub-Saharan Africa being CA [14]. In retrospect, the patient exhibited many red flags in echocardiography and ECGs that could have led to an earlier diagnosis.

Echocardiography

Transthoracic Echocardiography

This patient had transthoracic echocardiography (TTE) features that strongly indicated underlying CA, most importantly concentric left ventricular hypertrophy (LVH) reaching >15 mm, with the cut-off being >12 mm in CA. Other TTE findings that could indicate an underlying RCM that were present in this patient include long-term HFpEF before deterioration, distinct diastolic dysfunction, granular speckling, and biatrial dilatation [15]. Additional features can indicate RCM but were not seen in this patient, including pleural and pericardial effusions with LVH in addition to effusions in TTE having the highest specificity for underlying RCM small ventricles, raised filling pressures, and valve thickening [15].

As a diagnostic tool for CA, echocardiography is limited due to the absence of such findings in many patients. In addition, most of these findings can be seen in other cardiac diseases, such as chronic uncontrolled hypertension and hypertrophic obstructive cardiomyopathy (HOCM). For example, as well as being seen in CA, speckling has been described in numerous other conditions, including hypertensive heart disease, chronic kidney disease, HOCM, and Pompe’s disease [15].

Transesophageal Echocardiography

Transesophageal echocardiography (TEE) in CA is useful for demonstrating intracardiac thrombi, as was present in this patient, specifically in the left atrial appendage (LAA) and was spotted very early on in the disease before other significant echocardiographic features. Thrombi in CA are caused by both AF as well as reduced blood flow velocity in the LAA. This low blood flow velocity is due to impaired diastolic function of the left ventricle and impaired mechanical function of the dilated left atrium [16]. Feng et al. examined 116 patients with CA post-mortem and found intracardiac thrombi in 33% of the cases [17]. TEE, done more than once in this patient, allowed early visualization of an LAA thrombus, which later dissolved with incessant anticoagulation use, and was detected at a decreased LAA velocity of 27 cm/sec.

Speckle Tracking Echocardiography

CA patients often demonstrate relative preservation of apical function, leading to a “bull’s-eye” LVH pattern, as well as longitudinal deformation, with 93% sensitivity and 82% specificity [15]. When *eventually* performed, this patient had positive findings, with an apex:base ratio of >2.1, which, if found in a patient, helps distinguish cardiac amyloidosis from other causes of left ventricular hypertrophy [15].

Cardiac magnetic resonance

The superiority of CMR imaging in CA diagnosis lies in its ability to characterize myocardial tissue and the high precision of its measurements. CMR imaging typically employs cine imaging, native and post-contrast T1 mapping, and T2 mapping, as well as the administration of a gadolinium contrast agent. The net result is a highly specific assessment of wall thickness and function of, for example, the left ventricle and atrial size [16].

T1 Mapping

T1 depends on both intracellular and extracellular/interstitial factors. A very recent publication studied a large group of patients with suspected systemic amyloidosis, and although only applied to select individuals, it achieved high sensitivity (92%) and specificity (91%) [18]. The patient’s T1 SCOUT mapping showed nulling of blood pool before the myocardium after infusion of gadolinium-based contrast, the opposite of what is normally seen, plus a global epicardial-delayed enhanced lesion mainly affecting the lateral and anterior wall consistent with infiltrative cardiomyopathy, most likely CA.

T2 Mapping

Elevated transverse relaxation time, or T2, is specific for increased myocardial water content and free water, and is used as an index of myocardial edema. In CA, amyloid deposition and its toxic effect on cardiac cells is most likely the cause of myocardial edema and, thus, prolonged T2 [19]. The patient’s T2 CMR imaging actually did not show any myocardial edema.

Late Gadolinium Enhancement

The patient’s late gadolinium enhancement (LGE) showed severe asymmetrical septal hypertrophy, with extensive transmural heterogenous LGE involving basal, mid, and apical segments. These findings were congruous with CA, especially in the advanced stages of the disease [16].

Limitations of Cardiac Magnetic Resonance Imaging

Several factors can impede the use of contrast in CMR imaging, including age, sex (with extracellular volume distribution and hematocrit affecting T1 and T2, respectively), respiratory motion [16,18,19], claustrophobia, presence of a pacemaker/implantable cardioverter‐defibrillator, arrythmias [20], and renal dysfunction [16]. Nevertheless, clinicians are finding new ways to circumvent such limitations. It was even found that the presence of some limitations (e.g., rate-controlled AF) do not hinder the CMR imaging process, and important diagnostic data can still be acquired, as was the case for this patient, who had AF throughout CMR imaging.

Tc99m pyrophosphate single-photon emission computed tomography

Paired with hemato-oncological testing, cardiac PYP SPECT can differentiate AL-CA from ATTR-CA and other myocardial disorders without the need for biopsy. Endomyocardial biopsy (EMB) is considered the gold standard for the diagnosis of cardiac amyloidosis when combined with mass spectrometry. However, given the heterogeneity of CA, EMB may fail to diagnose the disease. Additionally, EMB is an invasive procedure and can lead to serious complications [21]. In our patient, cardiac PYP SPECT showed a cardiac/CL ratio of 1.27 (positive >1.5), with mild cardiac PYP uptake considered equivocal for ATTR-CA. However, as equivocal results could represent either AL-CA or early ATTR-CA, the distinction had to be made solely by hemato-oncological testing.

## Conclusions

While rapidly evolving diagnostic and management strategies for CA are seen, it still presents a diagnostic challenge in clinical medicine due to its perceived rarity and non-specific symptomatology. In this case review, certain red flags were brought up, which should have indicated further testing early on for underlying CA. A comparison between the roles of four imaging modalities is presented: echocardiography, STE, cardiac MRI, and cardiac Tc99m pyrophosphate single-photon emission computed tomography. The role of each modality in diagnosing CA was reported while highlighting their different advantages.

## References

[1] Benson MD, Buxbaum JN, Eisenberg DS (2018). Amyloid nomenclature 2018: recommendations by the International Society of Amyloidosis (ISA) nomenclature committee. Amyloid.

[2] Jamal F, Rosenzweig M (2021). Amyloidosis with cardiac involvement: identification, characterization, and management. Curr Hematol Malig Rep.

[3] Castiglione V, Franzini M, Aimo A (2021). Use of biomarkers to diagnose and manage cardiac amyloidosis. Eur J Heart Fail.

[4] Garcia-Pavia P, Rapezzi C, Adler Y (2021). Diagnosis and treatment of cardiac amyloidosis. A position statement of the European Society of Cardiology Working Group on Myocardial and Pericardial Diseases. Eur J Heart Fail.

[5] Martinez-Naharro A, Hawkins PN, Fontana M (2018). Cardiac amyloidosis. Clin Med (Lond).

[6] Timóteo AT, Rosa SA, Brás PG, Ferreira MJ, Bettencourt N (2022). Multimodality imaging in cardiac amyloidosis: state-of-the-art review. J Clin Ultrasound.

[7] Mohty D, Omer MH, Ahmad O, Alayary I, Alzahrani T, Damy T, Fadel B (2023). Transthyretin cardiac amyloidosis in Saudi Arabia and the Middle East: insights, projected prevalence and practical applications. Front Cardiovasc Med.

[8] Vaxman I, Gertz M (2020). When to suspect a diagnosis of amyloidosis. Acta Haematol.

[9] Gertz MA (2022). Cardiac amyloidosis. Heart Fail Clin.

[10] Sabbour H, Hasan KY, Al Badarin F, Alibazoglu H, Rivard AL, Romany I, Perlini S (2021). From clinical clues to final diagnosis: the return of detective work to clinical medicine in cardiac amyloidosis. Front Cardiovasc Med.

[11] Ash S, Shorer E, Ramgobin D (2021). Cardiac amyloidosis - a review of current literature for the practicing physician. Clin Cardiol.

[12] Garcia-Pavia P, Rapezzi C, Adler Y (2021). Diagnosis and treatment of cardiac amyloidosis: a position statement of the ESC Working Group on Myocardial and Pericardial Diseases. Eur Heart J.

[13] Trachtenberg BH, Kamble RT, Rice L (2019). Delayed autologous stem cell transplantation following cardiac transplantation experience in patients with cardiac amyloidosis. Am J Transplant.

[14] Muchtar E, Blauwet LA, Gertz MA (2017). Restrictive cardiomyopathy: genetics, pathogenesis, clinical manifestations, diagnosis, and therapy. Circ Res.

[15] Moody WE, Turvey-Haigh L, Knight D (2023). British Society of Echocardiography guideline for the transthoracic echocardiographic assessment of cardiac amyloidosis. Echo Res Pract.

[16] Waldmeier D, Herzberg J, Stephan FP, Seemann M, Arenja N (2022). Advanced imaging in cardiac amyloidosis. Biomedicines.

[17] Feng D, Edwards WD, Oh JK (2007). Intracardiac thrombosis and embolism in patients with cardiac amyloidosis. Circulation.

[18] Baggiano A, Boldrini M, Martinez-Naharro A (2020). Noncontrast magnetic resonance for the diagnosis of cardiac amyloidosis. JACC Cardiovasc Imaging.

[19] O'Brien AT, Gil KE, Varghese J, Simonetti OP, Zareba KM (2022). T2 mapping in myocardial disease: a comprehensive review. J Cardiovasc Magn Reson.

[20] Assomull RG, Pennell DJ, Prasad SK (2007). Cardiovascular magnetic resonance in the evaluation of heart failure. Heart.

[21] Li W, Uppal D, Wang YC, Xu X, Kokkinidis DG, Travin MI, Tauras JM (2021). Nuclear imaging for the diagnosis of cardiac amyloidosis in 2021. Diagnostics (Basel).

